# Does Opium Dependency Affect the Pattern of Involvement in Laryngeal Cancer?

**Published:** 2016-11

**Authors:** Peyman Dabirmoghaddam, Ali Karimian Taheri, Hosein Ghazavi, Shaghayegh Ebrahimnejad, Zahra Karimian

**Affiliations:** 1 *Department of Otorhinolaryngology, Tehran University of Medical Science, Tehran, Iran.*; 2 *Student Research Committee, School of Nursing and Midwifery, Shahroud University of Medical Sciences, Shahroud, Iran*

**Keywords:** Iran, Laryngeal cancer, Opium

## Abstract

**Introduction:**

Laryngeal cancer is the second most common cancer involving the respiratory tract, second only to lung cancer. Previous studies have shown opium dependency to be a possible risk factor for laryngeal cancer. Opium consumption is a major problem in some parts of the world. The aim of this study was to investigate the effect of opium consumption on the pattern of involvement in laryngeal cancer.

**Methods and materials:**

In this analytical cross-sectional study, 44 patients diagnosed with laryngeal cancer (41 male and three female) were studied. Data were collected using a questionnaire, physical examination, and paraclinical studies. Patients were defined as opium dependent based on the Diagnostic and Statistical Manual of Mental Disorders (Fourth Edition) (DSM-IV) criteria and duration of opium consumption.

**Results:**

Patients were categorized into two groups: opium-dependent (32.6%) and non-opium-dependent (67.4%) patients. The average age of the patients was 58.5±3.2 years in the opium-dependent group and 60.7±2.8 years in the non-opium-dependent group (P=non-significant). An analysis of the pattern of involvement in each group showed glottis involvement in 56%, subglottis involvement in 8%, supraglottis involvement in 32%, and hypopharyngeal involvement in 4% of the opium-dependent group compared with glottis involvement in 55.9%, subglottis involvement in 11.8%, supraglottis involvement in 32.4%, and hypopharyngeal involvement in none of the non-opium-dependent group (P=non-significant).

**Conclusion:**

There was a poor correlation between opium dependency and pattern of involvement in laryngeal cancer. Opium dependency did not lead to the development of laryngeal cancer at a younger age, based on our study.

## Introduction

Laryngeal cancer accounts for 2.2% of all cancers among men and 0.4% among women. It is the second most common cancer involving the respiratory tract, second only to lung cancer (1). In cases of malignancy, the laryngeal epithelium shows local thickening with white or erythematous discoloration due to hyperkeratosis. Laryngeal cancer cells metastasize slowly while the tumor enlarges, and the disease usually involves regional tissues and lymph nodes of the neck. Regional metastasis may be present in up to half of cases on presentation, although distant metastasis is not common even in advanced-stage disease (2). The symptoms of laryngeal squamous cell carcinoma (SCC) depend on the site from which the primary tumor originates. The cardinal symptom of glottic SCC is dysphonia. Supraglottic tumors may cause dysphonia, which often manifests as an alteration in vocal resonance, and may also cause dysphagia, odynophagia, otalgia, stridor, dyspnea, and hemoptysis. SCC of the subglottis is often seen with advanced-stage disease, and dyspnea and stridor are the most common symptoms of subglottic SCC (3).

The larynx is divided into three sections. The supraglottis is the area above the vocal cords containing the epiglottis, which closes off the larynx on swallowing to prevent transit of food and fluids into the lungs. The glottis is the area containing the vocal cords, while the subglottis is the area below the vocal cords. Laryngeal tumors involve different sites of the larynx, including the glottis, supraglottis, and subglottis. In the United States (US), laryngeal SCC occurs in the glottis more frequently than in the supraglottis. Subglottic SCC is rare (3).

The prevalence of laryngeal cancer varies across different regions of the world. Studies suggest that it occurs most frequently in South America and Mediterranean countries, while the lowest incidence is found in Finland (4).

Many risk factors are known to contribute to the occurrence of laryngeal cancer. For example, genetic background, diet, asbestos exposure, increased age, infrequent consumption of fruits and vegetables, and occupation can be potential risk factors for laryngeal cancer. Tobacco and alcohol consumption are the two primary risk factors for cancer of the larynx (5–9). In addition, Saltor et al. showed that contact with materials such as coal, wood, gas, and polluting emissions in industrial environments can contribute in the pathogenesis of this cancer (10).

 A study by Mousavi et al. revealed that opium dependency is not only an independent possible risk factor for laryngeal cancer but also significantly increases the likelihood of developing of the disease at a younger age (11). Consistent findings were reported by Damghani et al. in a study which showed that opium was a risk factor for laryngeal cancer (1). When opium is smoked, it produces a large number of mutagenic agents such as polycyclic aromatic hydrocarbons (PAHs), which are known to be potent carcinogens (12). As a major group of organic compounds, PAHs can induce DNA damage and increase cancer susceptibility (13).

The prevalence of opioid consumption varies from 0.1–2% in the world. Opioids abuse is the most common form of drug abuse in Iran. In 2003, it was reported that approximately 1.2 million addicts live in Iran, and that this number may increase by 8% each year (1). In a study by Ziaodini et al., prevalence of opium use was 7.1% among male students, and a prevalence of 8.6% was reported in a study by Yaghobi et al. (14,15). Because of the high incidence of laryngeal cancer and the association between opium dependency and laryngeal cancer, this study was designed to investigate the effect of opium dependency on the pattern of involvement in laryngeal cancer among patients attending Imam Khomeini Hospital, Tehran, between January 2012 and January 2013.

## Materials and Methods

In this analytical cross-sectional study, we evaluated 44 patients diagnosed with laryngeal cancer attending Imam Khomeini Hospital, Tehran between January 2012 and January 2013. Data collection was conducted by means of a questionnaire designed by the researcher which assessed four categories of data: 1) demographic data; 2) risk factors and symptoms of laryngeal cancer; 3) pattern of involvement in laryngeal cancer; 4) pathologic, radiographic findings, and staging of laryngeal cancer. Data on cigarette smoking (measured by number of cigarettes smoked per day and duration of smoking), alcohol consumption, and addiction to other agents was also recorded. Pattern of involvement in laryngeal cancer was recorded as follows: involvement of either of four sites including the glottis, subglottis, supraglottis, and hypopharynx region. The questionnaire met three main psychometric criteria, including responsiveness, reliability, and the validity required for every standard questionnaire.

Patients were excluded from this study if their diagnosis of laryngeal cancer was uncertain, if a full medical history was not available, or if clinical, radiographic, or pathologic data were not available. Laryngeal cancer diagnosis was achieved through an otorhinolaryngologist visit and was based upon clinical examination and paraclinical data. Opium dependency was determined through the Diagnostic and Statistical Manual of Mental Disorders (Fourth Edition) (DSM-IV), prepared by the Task Force on Nomenclature and Statistics of the American Psychiatric Association (APA). 

DSM-IV is the APA’s official manual of mental disorders and provides detailed descriptions of categories of disorders as well as diagnostic criteria (16). All patients who consumed opium for at least 5 years and fulfilled DSM-IV criteria for opium dependency were considered opium dependent. Written informed consent was obtained from patients after the aim of study was clearly explained. Patients were reassured that their data would remain secret. Data obtained using the questionnaire was recorded, and statistical analysis was performed using SPSS software (version 17.0 for windows). Descriptive statistics were used to determine the frequency and description of the data. Associations between variables were assessed using analytical statistics. A p-value less than 0.05 was considered statistically significant.

## Results

Forty-four patients diagnosed with laryngeal cancer were included in this study. The majority of patients (88.6%) were male and were not officially employed (62%). Table 1 summarizes the age distribution of patients, showing that most were between 60 and 69 years of age. The majority of patients in our study were smokers (83.7%), with only seven patients who did not smoke. Previous studies have shown a marked correlation between smoking and laryngeal cancer (17-19). In total, 54.8% of patients presented with dysphonia, 28.6% with a breathy voice, 11.9% with hoarseness, 2.4% with dysphagia, and 2.4% with dyspnea. Vocal cord motion was normal in 84.2% of patients. Based on TNM staging, the size of most specimens was 1–7 centimeters in length, and 89.7% showed no lymph node involvement. No metastasis was reported, and most pathology reports revealed SCC. Table 2 shows a comparison of research variables between the opium-dependent and non-opium-dependent groups. The difference between the two groups was not statistically significant. A comparison of the pattern of involvement between the opium-dependent and non-opium-dependent groups is shown in Table 3. The difference observed between the two groups was not statistically significant. The glottis area was the most involved site in both groups.

**Table 1:** Age distribution of patients

**Age group **	**Number**	**Percentage**
<39 years	3	6.8
40–49 years	3	6.8
50–59 years	14	31.8
60–69 years	15	34.1
≥70 years	9	20.5
Total	44	100

**Table 2:** Comparison of research variables between opium-dependent and non-opium-dependent groups

**Variable**		**Opium-dependent**		**Non-opium-dependent**		**P-value**
		Number	Percent	Number	Percent	
Sex	Male	13	100	28	90.3	0.318
	Female	0	0	3	9.7	
Cigarette smoking	Yes	12	92.9	25	80.6	0.239
	No	1	7.1	6	19.4	
Age average		58.5±3.32		60.7±2.28		0.65

**Table 3:** Comparison of pattern of involvement by laryngeal cancer between opium-dependent and non-opium-dependent groups.

**Site of** **involvement**	**Opium-dependent**	**Non-opium-dependent**	**P-value**
Glottis	56%	55.9%	0.668
Subglottis	8%	11.8%	
Supraglottis	32%	32.4%	
Hypopharynx	4%	0%	
Total	100%	100%	

## Discussion

Forty-four patients diagnosed with laryngeal cancer were enrolled in this study. The age range of patients was 60–69 years (mean, 60.02±12.1 years). Various studies suggest an age range of 60–70 years is most common for presentation of laryngeal cancer (1,11). However, one of our patients, a smoker but not opium dependent, was 31 years of age.

Three of our subjects were female. It is known that the incidence of laryngeal cancer is greater among men compared with women, although changing lifestyles, including cigarette and alcohol consumption, might lead to a change in this proportion (20).

The majority of our patients smoked. The strong association between smoking and laryngeal cancer has been shown in previous studies (17-19), with cigarette smoking known to be the most important known risk factor for laryngeal cancer. Saki et al. showed that the most common predisposing factor in the development of laryngeal cancer is smoking, and a diagnosis of laryngeal cancer is unusual for non-smokers. They also reported that smoking cessation is the best way to prevent cancer of the larynx (8).

Based on previous studies, opium is a known carcinogenic agent (21-23) however, there are few studies available regarding the association between opium dependency and laryngeal cancer (11). In total, 32.6% of our subjects were opium-dependent.

The mean age for diagnosis of laryngeal cancer in opium-dependent and non-opium-dependent patients in our study was 58.5±3.32 years and 60.7±2.28 years, respectively. These findings suggest that opium dependency is not associated with the age of diagnosis of laryngeal cancer. This finding contradicts the results of Mousavi et al. who showed that the age of incidence of laryngeal cancer was 10.4 years lower in opium-dependent patients than in non-opium-dependent patients )^11^).

There are some limitations in our study which must be noted. For example, some environmental factors such as diet, alcohol consumption, and occupation may modulate the carcinogenic effects of opium. Therefore, we suggest that the future studies be performed using a larger sample size to take into account environmental factors. Further studies with a larger sample size would also help to elucidate the exact relationship between opium dependency and age at presentation of laryngeal cancer.

## Conclusion

We found a poor correlation between opium dependency and pattern of involvement in laryngeal cancer. Opium dependency did not lead to the development of laryngeal cancer at a younger age, based on our study. Further studies with a larger sample size are required to confirm these findings.

## References

[1] Damghani M, Bazrafshani M, Mirshekari T (2012). The Prevalence of P53 Mutations in Laryngeal Cancer in Kerman. J Kerman Uni Med Sci.

[2] Azarhosh R, Taziki M (2003). Metastatic cutaneous laryngeal cancer to form abscess, a case report. J Gorgan Uni Med Sci.

[3] Flint PW, Haughey BH, Robbins KT, Thomas JR, Niparko JK, Lund VJ (2014). Cummings otolaryngology–head and neck surgery.

[4] Sarafraz M, Nik Akhlagh S, Ghazipour A, Saki N, Ahmadi Kh (2007). Comparative evaluation of laryngeal cancer risk factors in elderly and non-elderly patients. Med Sci J.

[5] Shan'gina OV, Sdvizhkov AM, Finkel'shtern MR, Brennan P, Boffetta P, Zaridze DG (2007). Risk factors of laryngeal cancers in central and Eastern Europe. Vopor Onkol.

[6] Risch A, Ramroth H, Raedts V, Rajaee-Behbahani N, Schmezer P, Bartsch H, Becher H, Dietz A (2003). Laryngeal cancer risk in Caucasians is associated with alcohol and tobacco consumption but not modified by genetic polymorphisms in class I alcohol dehydrogenases ADH1B and ADH1C, and glutathione-S-transferases GSTM1 and GSTT1. Pharmacogenetics and Genomics.

[7] Muscat JE, Wynder EL (1992). Tobacco, alcohol, asbestos, and occupational risk factors for laryngeal cancer. Cancer.

[8] Zheng W, Blot WJ, Shu XO, Gao YT, Ji BT, Ziegler RG, Fraumeni JF (1992). Diet and other risk factors for laryngeal cancer in Shanghai, China. American Journal of Epidemiology.

[9] De Stefani E, Correa P, Oreggia F, Leiva J, Rivero S, Fernandez G, Deneo‐Pellegrini H, Zavala D, Fontham E (1987). Risk factors for laryngeal cancer. Cancer.

[10] Sartor SG, Eluf-Neto J, Travier N, Wünsch Filho V, Arcuri AS, Kowalski LP (2007). Occupational risks for laryngeal cancer: a case control study. Cad Saúde Pública.

[11] Mousavi MR, Damghani MA, Haghdoust AK, Khamesipour A (2003). Opium and Risk of Laryngeal Cancer. Laryngoscope.

[12] Kamangar F, Chow WH, Abnet CC, Dawsey SM (2009). Environmental causes of esophageal cancer. Gastroenterol Clin N Am.

[13] Xue W, Warshawsky D (2005). Metabolic activation of polycyclic and heterocyclic aromatic hydrocarbons and DNA damage: a review. Toxicol App Pharmacol.

[14] Zaiadinin et al (2006). The prevalence of drug abuse and addiction and related factors in the final year students. Kerman Med Uni J.

[15] Yaghobi (2012). Epidemiology, causes of addiction among the students of Mazandaran University of Medical Sciences. National Conference on Addiction.

[16] Schacter DS, Gilbert DT, Wegner DM (2011). Psychology.

[17] Saki N, Saki GH, Masomi A, Rahim F, Emad N (2011). Assess the impact of smoking on laryngeal cancer in Khuzestan. J Res Jondishapour.

[18] Vassileiou A, Vlastarakos PV, Kandiloros D, Delicha E, Ferekidis E, Tzagaroulakis A, Nikolopoulos TP (2012). Laryngeal cancer: smoking is not the only risk factor. B-ENT.

[19] Menach P, Oburra HO, Patel A (2012). Cigarette smoking and alcohol ingestion as risk factors for laryngeal squamous cell carcinoma at Kenyatta national hospital, Kenya. Clin Med Insights Ear Nose Throat.

[20] Parker SL et al (1996). Cancer statistics. Cancer.

[21] Ghavamzadeh A, Moussavi A, Jahani M, Rastegarpanah M, Iravani M (2001). Esophageal cancer in Iran. Semin Oncol.

[22] Sadjadi A, Derakhshan MH, Yazdanbod A, Boreiri M, Parsaeian M (2014). Neglected role of hookah and opium in gastric carcinogenesis: a cohort study on risk factors and attributable fractions. Int J Cancer.

[23] Shakeri R, Malekzadeh R, Etemadi A, Nasrollahzadeh D, Aghcheli K (2013). Opium: an emerging risk factor for gastric adenocarcinoma. Int J Cancer.

